# Predictive Factors for Impaired Mental Health among Medical Students during the Early Stage of the COVID-19 Pandemic in Morocco

**DOI:** 10.4269/ajtmh.20-1302

**Published:** 2020-11-17

**Authors:** Hajar Essangri, Maria Sabir, Amine Benkabbou, Mohammed Anass Majbar, Laila Amrani, Abdelilah Ghannam, Brahim Lekehal, Raouf Mohsine, Amine Souadka

**Affiliations:** 1 Surgical Oncology Department, National Institute of Oncology, University Mohammed V in Rabat, Rabat, Morocco;; 2 Arrazi University Psychiatric Hospital, University Mohammed V in Rabat, Rabat, Morocco;; 3 Equipe de Recherche en Oncologie Translationnelle (EROT), Faculty of Medicine and Pharmacy, University Mohamed V in Rabat, Rabat, Morocco;; 4 Anesthesia and Intensive Care Department, National Institute of Oncology, University Mohammed V in Rabat, Rabat, Morocco;; 5 Department of Academic Affairs, Faculty of Medicine and Pharmacy, University Mohammed V in Rabat, Rabat, Morocco

## Abstract

The COVID-19 pandemic has great consequences on mental health. We aimed to assess medical students’ psychological condition and influencing factors as a baseline evidence for interventions promoting their mental wellbeing. We conducted an online survey from April 8 to April 18, 2020 to examine the mental health of medical students by the nine-item Patient Health Questionnaire, seven-item Generalized Anxiety Disorder Scale, seven-item Insomnia Severity Index, and six-item Kessler psychological distress scale. Factors associated with mental health outcomes were identified by multivariable logistic regression analysis. Five hundred forty-nine students completed the survey; 341 (62.3%), 410 (74.6%), 344 (62.6%), and 379 (69%) reported anxiety, depression, insomnia, and distress, respectively. Female students, living in high COVID-19 prevalence locations, more than 25 days confinement, psychiatric consult history, and being in a preclinical level of studies had higher median scores and severe symptom levels. Multivariable logistic regression showed female gender as a risk factor for severe symptoms of anxiety (odds ratio [OR]: 1.653; 95% CI: 1.020–2.679; *P* = 0.042), depression (OR: 2.167; 95% CI: 1.435–3.271; *P* < 0.001), insomnia (OR: 1.830; 95% CI: 1.176–2.847; *P* = 0.007), and distress (OR: 1.994; 95% CI: 1.338–2.972; *P* = 0.001); preclinical level of enrollment as a risk factor for depression (OR: 0.679; 95% CI: 0.521–0.885; *P* = 0.004), insomnia (OR: 0.720; 95% CI: 0.545–0.949; *P* = 0.02), and distress (OR: 0.650; 95% CI: 0.499–0.847; *P* = 0.001), whereas living in high COVID-19 prevalence locations was a risk factor for severe anxiety (OR: 1.628; 95% CI: 1.090–2.432; *P* = 0.017) and depression (OR: 1.438; 95% CI: 1.002–2.097; *P* = 0.05). Currently, medical students experience high levels of mental health symptoms, especially female students, those at a preclinical level and living in regions with a high prevalence of COVID-19 cases. Screening for mental health issues, psychological support, and long-term follow-up could alleviate the burden and protect future physicians.

## INTRODUCTION

As of May 10, 2020, the COVID-19 pandemic has been confirmed in 210 countries worldwide, with a total of 4,117,098 infected people^1^ and a crude mortality ratio of 3–4%. The COVID-19 pandemic has brought not only the risk of death from infection but also unbearable psychological pressure. The toll that coronavirus is taking on mental health is extending beyond infected people, and the emanating mental health pandemic will last further.

Whereas the mental health consequences of past infectious outbreaks were mainly sequela of the disease itself,^2^ several studies discuss the dominant emotional response to the COVID-19 outbreak^3^ and the current health anxiety^4^ that is driven by misinformation. This has called for the immediate collection of high-quality data on the mental health effects of the COVID-19 pandemic.^5^ Accordingly, the psychological impact of the pandemic was reported on the general population, children, and older adults.^6–8^

Patients and healthcare workers are similarly vulnerable to the emotional impact of coronavirus.^9^ Patients with COVID-19 infections are considered at risk of mental health issues as they face anxiety, uncertainty about the future of their health, and feel stigmatized.^10^ Likewise, healthcare professionals are being subjected to change, uncertainty, stress, and isolation, in addition to an emotional distress of exceptional intensity, and need to be protected.^11,12^ This is manifesting in up to 70% of healthcare workers suffering from psychological distress.^13^

The COVID-19 crisis puts medical students under considerable psychological pressure as well. In addition to being submitted to similar constraints as the general population, they face brutal changes in teaching methods, uncertainty about their academic future, and some of them even participate in the fight.^14,15^ As medical students are known for presenting higher rates of anxiety, depression, and burnout,^16,17^ these new circumstances may put their mental well-being at risk. During this pandemic, the mental health of healthcare professionals was assessed through depression, anxiety, insomnia, and distress. In a similar manner, we aimed to assess medical students’ psychological conditions and factors influencing these conditions as baseline evidence for interventions to promote their mental well-being.

## MATERIALS AND METHODS

Our study followed the American Association for Public Opinion Research reporting guidelines for survey studies and the Strengthening the Reporting of Observational studies in Epidemiology directive guidelines for observational studies.^18,19^

### Study design.

We investigated and analyzed the mental health status of medical students during the pandemic for the following purposes: to evaluate the mental situation of medical students during the pandemic and to provide a theoretical basis for psychological interventions with medical students.

This study is a cross-sectional online survey conducted from April 8 to April 18, 2020, with Morocco having at these dates 1,242 and 2,685 confirmed cases (Supplemental Appendix 1). Before conducting the study, a pilot test was conducted on five participants to examine their understanding of the questions. The results of the pilot study were not included in the analysis. The survey was distributed through social media channels, student board pages, and institutional emails. The questionnaires were anonymous to ensure the confidentiality and reliability of data, and consent was provided at the beginning of the survey.

### Participants and sampling.

We targeted medical students at different levels of training whether at a preclinical (first and second year) or clinical level (third to seventh year), alongside graduates before the beginning of their residency. To understand interregional differences in the mental health impact of the pandemic, medical students from the seven faculties were invited to participate, with an estimated total of 13,550 students. The target sample size of participants was estimated according to the formula *n* = (*z*^2^ × *p* × [1 − *p*]/*e*^2^)/{1 + (*z*^2^ × *p* × [1 − *p*]/[*e*^2^ × *N*])} with *e =* 95 and *Z* = 1.96, and the minimum required number of responses was 374. Participants were categorized according to the same regional distribution used to document the number of COVID-19 cases by the Ministry of Health.

### Outcomes and covariates/survey.

We assessed the mental health of medical students during the COVID-19 outbreak using structured questionnaires. We choose to assess specific symptoms of depression, anxiety, and insomnia in our participants. Accordingly, we used the French validated versions of the nine-item Patient Health Questionnaire (PHQ-9; range, 0–27),^20^ the seven-item Generalized Anxiety Disorder Scale (GAD-7), the seven-item Insomnia Severity Index (ISI; range, 0–28),^21^ and the Kessler six nonspecific psychological distress scale (K-6; range, 0–24).^22^

Patient Health Questionnaire-9 is a nine-item self-rating instrument, with each item representing one of the Diagnostic and Statistical Manual of Mental Disorders, fourth Edition criteria for a depressive episode (anhedonia, depressed mood, sleep problems, feeling tired, change in appetite, negative self-evaluation, concentration problems, psychomotor changes, and suicidality). Each item can be scored according to a four-item Likert scale ranging from zero (not at all) to three (nearly every day), according to the frequency of experiencing difficulties in the respective area in the previous 2 weeks.^23^

The GAD-7 includes seven items based on seven core symptoms and inquires the frequency with which respondents suffered from these symptoms within the last 2 weeks.^24^ Respondents report their symptoms using a four-item Likert rating scale ranging from zero (not at all) to three (almost every day).

The insomnia severity evaluates 1) the severity of sleep onset (initial), 2) sleep maintenance (middle), 3) early morning awakening (terminal) problems, 4) satisfaction with current sleep pattern, 5) interference with daily functioning, 6) noticeability of impairment attributed to the sleep problem, and 7) level of distress caused by the sleep problem.^25^

The Kessler-6 instrument on the other hand evaluates psychological distress by asking: “During the past 30 days, how often did you feel 1) nervous? 2) hopeless? 3) restless or fidgety? 4) so depressed that nothing could cheer you up? 5) that everything was an effort? 6) worthless?” Possible responses are “none of the time,” “a little of the time,” “some of the time,” “most of the time,” and “all of the time,” and the scoring is on a five-point Likert scale.^26^

The total scores of these measurement tools were interpreted as suggested by their authors with PHQ-9, normal (0–4), mild (5–9), moderate (10–14), and severe (15–21) depression^23^; GAD-7, normal (0–4), mild (5–9), moderate (10–14), and severe (15–21)^27^ anxiety; ISI, normal (0–7), subthreshold (8–14), moderate (15–21), and severe (22–28) insomnia^25^; and K-6, no psychological distress (0–7), moderate (8–12), and severe (13–24) psychological distress.^13,26^ The cutoff scores for detecting symptoms of major depression, anxiety, insomnia, and distress were 10, 10, 15, and 13, respectively.^13,23,25–27^

The study instrument comprised a structured questionnaire packet that inquired demographic information including age (categorized according to the mean: ≤ 22 or > 22 years), gender, region, and place of residence (the geographic location of participants according to whether they are in a region with low or high prevalence of COVID-19 cases was also reported), relationship status (single or married), having children, smoking habits, and history of psychiatric consult. As medical studies in Morocco extend to a 7–8-year period, we categorized enrollment levels according to preclinical (first and second years), early (third to sixth year), and late (seventh and graduates before residency start) clinical levels in medical studies. Respondents were also asked whether they were in confinement or not and for how long. The confinement period was categorized according to the mean period of 25 days.

### Statistical analysis.

An analysis of descriptive statistics was conducted to illustrate the demographic and other selected characteristics of the respondents.

Continuous variables were presented as mean values ± SD or as medians with interquartile ranges (IQRs). Categorical variables were expressed as frequencies and percentages. The nonparametric Mann–Whitney *U* test and Kruskal–Wallis test were applied to compare the severity of each symptom between two or more groups. The chi-square test was used to compare the severity of symptoms according to the different categories, whereas the median scores were compared by nonparametric tests. A multivariate logistic regression analysis including all statistically significant variables for mental health symptoms was built to identify the predictive factors. The estimates of the strengths of associations were demonstrated by the odds ratio (OR) with a 95% CI. A stepwise binary logistic regression model was built to identify the predictive factors of symptoms of depression, anxiety, insomnia, and distress, and the resulting association was presented as ORs and 95% CIs. A two-tailed *P* < 0.05 was considered statistically significant. Data were analyzed with SPSS version 25.00 (SPSS Inc., Chicago, IL).

### Ethical considerations.

All participants voluntarily gave their informed consent to participate in the study after being informed about its purpose. This study was approved by the Ethics Committee of the faculty of medicine of Rabat, Morocco.

## RESULTS

### Demographic characteristics.

The demographic and selected characteristics of the study population are shown in Table 1.

**Table 1:** Demographic characteristics of participants

Characteristic	No	%
Age (years)		
Mean age	22 ± 3	–
> 22	208	37.9
≤ 22	341	62.1
Gender		
Male	143	26
Female	406	74
Status		
Single	448	81.6
Married	101	18.4
Children		
No	537	97.8
Yes	12	2.2
Study level		
Preclinical	170	31
Early clinical	261	47.5
Late clinical	118	21.5
Tobacco use		
No	518	94.4
Yes	31	5.6
History of psychiatric consult		
No	442	80.5
Yes	107	19.5
Confinement (days)		
Not confined	66	12
≤ 25	220	40.1
> 25	263	47.9
Region		
High prevalence of COVID cases (Casablanca-Marrakech)	180	32.8
Low prevalence of COVID cases	365	66.5

Among the sample of 549 medical students who responded to the questionnaire, 406 (74%) were women, 448 (81.6%) were single, and 107(19.5%) had a history of psychiatric consult. One hundred eighty participants (32.8%) lived in a region with high prevalence of diagnosed COVID-19 cases and 263 (47.9%) had been quarantined for more than 25 days. The mean age was 22 ± 3 years (Table 1).

### Severity of measurements and associated factors.

Table 2 shows how the mental health of medical students was affected to varying degrees during the outbreak.

**Table 2:** Severity categories of depression, anxiety, insomnia, and distress measurements in total cohort and subgroups

	Total, *N* (%)	Location no. (%)			Confinement no. (%)				History of psychiatric consult no. (%)			Gender no. (%)			Level no. (%)			
		Low	High	*P*-value	No confinement	< 25 days	> 25 days	*P*-value	No	Yes	*P*-value	Male	Female	*P*-value	Preclinical	Early clinical	Late clinical	*P*-value
Severity category		Prevalence Prevalence																
GAD (anxiety)																		
Normal (0–4)	207 (37.7)	150 (41.1)	56 (31.1)	0.003	27 (41)	93 (42.3)	87 (42)	0.04	173 (39.1)	34 (31.8)	< 0.001	71 (49.7)	136 (33.5)	0.005	51 (30)	97 (37.2)	59 (50)	0.027
Mild (5–9)	201 (36.6)	134 (36.7)	66 (36.7)		18 (27.3)	86 (42.8)	97 (48.3)		165 (37.3)	36 (33.6)		46 (32.2)	155 (38.2)		74 (43.5)	90 (34.5)	37 (31.4)	
Moderate (10–14)	87 (15.8)	56 (15.3)	29 (16.1)		15 (22.7)	24 (27.6)	48 (55.2)		73 (16.5)	14 (13.1)		17 (11.9)	70 (17.2)		27 (15.9)	45 (17.2)	15 (12.7)	
Severe (15–21)	54 (9.8)	25 (6.8)	29 (16.1)		6 (9.1)	17 (31.5)	31 (57.4)		31 (7)	23 (21.5)		9 (6.3)	45 (11.1)		18 (10.6)	29 (11.1)	7 (5.9)	
Insomnia Severity Index (insomnia)																		
Normal (0–7)	205 (37.3)	145(39.7)	58 (32)	0.089	31 (47)	88 (40)	86 (32.7)	0.145	172 (38.9)	33 (30.8)	0.248	71 (49.7)	134 (33)	0.001	55 (32.4)	90 (34.5)	60 (50.8)	0.007
Subthreshold (8–14)	158 (28.8)	108 (29.8)	49 (27.2)		18 (27.3)	67 (30.5)	73 (27.8)		123 (27.8)	35 (32.7)		38 (26.6)	120 (29.6)		44 (25.9)	82 (31.4)	32 (27.1)	
Moderate (15–21)	147 (26.8)	91 (24.9)	55 (30.6)		14 (21.2)	53 (24.1)	80 (30.4)		119 (26.9)	28 (26.2)		30 (21)	117 (28.8)		55 (32.4)	69 (26.4)	23 (19.5)	
Severe (22–28)	39 (7.1)	21 (5.8)	18 (10)		3 (4.5)	12 (5.5)	24 (9.1)		28 (6.3)	11 (10.3)		4 (2.8)	35 (8.6)		16 (9.4)	20 (7.7)	3 (2.5)	
Patient Health Questionnaire-9 (depression)																		
Normal (0–4)	139 (25.3)	107 (29.3)	31 (17.2)	0.003	23 (34.8)	63 (28.6)	53 (20.2)	0.011	117 (26.5)	22 (20.6)	0.026	49 (34.3)	90 (22.2)	< 0.001	28 (16.5)	70 (26.8)	41 (34.7)	0.002
Mild (5–9)	159 (29)	107 (29.3)	50 (27.8)		19 (28.8)	64 (29.1)	76 (28.9)		124 (28.1)	35 (32.7)		49 (34.3)	110 (27.1)		49 (28.8)	69 (26.4)	41 (34.7)	
Moderate (10–14)	118 (21.5)	76 (20.8)	41 (22.8)		10 (15.2)	54 (24.5)	54 (20.5)		103 (23.3)	15 (14)		25 (17.5)	93 (22.9)		43 (25.3)	56 (21.5)	19 (16.1)	
Severe (15–21)	133 (24.2)	75 (20.5)	58 (32.2)		14 (21.2)	39 (17.7)	80 (30.4)		98 (22.2)	35 (32.7)		20 (14)	113 (27.8)		50 (29.4)	66 (25.3)	17 (14.4)	
Kessler (psychological distress)																		
No distress (0–7)	170	190 (51.6)	76 (42)	0.016	33 (50)	122 (55.5)	111 (42)	0.023	222 (50.2)	44 (41.1)	0.023	88 (61.5)	178 (43.5)	< 0.001	68 (40)	122 (46.7)	76 (64.4)	0.001
Moderate (8–12)	261	107 (29.1)	51 (28.2)		21 (31.8)	59 (26.8)	78 (29.7)		130 (29.4)	28 (26.2)		39 (27.3)	119 (29.3)		63 (37.1)	72 (27.5)	23 (19.5)	
Serious (> 13)	118	71 (19.3)	54 (29.8)		12 (18.2)	39 (17.7)	74 (28.1)		90 (20.4)	35 (3é.7)		16 (11.2)	109 (26.8)		39 (22.9)	67 (25.7)	19 (16.1)	

A substantial number of respondents were positive for symptoms of anxiety (341, 62.3%), depression (410, 74.6%), insomnia (344, 62.6%), and psychological distress (379, 69%).

Factors associated with higher median scores and severe symptom levels included female gender, living in high COVID-19 prevalence locations, history of more than 25 days confinement, history of psychiatric consultation, and history of preclinical level of studies (compared with early and late clinical level). Accordingly, severe anxiety in those confined for less or more than 25 days was observed in 17 (31.5%) and 31 (57.4%) of the cases, respectively (*P* = 0.04); severe insomnia in male and women participants was four (2.8%) and 35 (8.6%) (*P* = 0.001), respectively; severe depression in medical students at a preclinical level compared with those at an early or late clinical level was positive in 50 (29.4%), 66 (25.3%), and 17 (14.4%) of the cases (*P* = 0.002); and serious psychological distress was diagnosed in 71 (19.3%) and 54 (29.8%) according to whether they were in a location with low or high prevalence of COVID-19 cases (*P* = 0.016; Table 2).

### Scores of measurements and associated factors.

Table 3 shows the relationship between the demographic variables of medical students and depression, anxiety, insomnia, and psychological distress.

**Table 3:** Scores of depression, anxiety, insomnia, and distress measurements in total cohort and subgroups

	Total median score (interquartile range)	Location			Confinement				History of psychiatric consult			Gender			Level			
Scale		Low prevalence	High prevalence	*P*-value	No confinement	< 25 days	> 25 days	*P*-value	No	Yes	*P*-value	Male	Female	*P*-value	Preclinical	Early clinical	Late clinical	*P*-value
GAD (anxiety)	6.0 (3.0–10.0)	6 (3.0–9.0)	7 (4.0–12.0)	< 0.001	5.0 (3.0–10.0)	5.5 (3.0–8.75)	6.0 (4–11)	0.026	6.0 (3.0–9.0)	7.0 (4.0–13.0)	0.004	5.0 (2.0–8.0)	6.0 (3.0–10.0)	0.001	6.0 (4.0–10.0)	6.0 (3.0–10.0)	4.5 (2.0–8.25)	0.019
Insomnia Severity Index (insomnia)	11.0 (5.0–7.0)	10.0 (5.0–10)	12.0 (5.0–18.0)	0.004	9.0 (4.0–15)	9.5 (5.0–16.0)	12.0 (5.0–18.0)	0.013	10.0 (5.0–16.0)	12.0 (6.0–18.0)	0.142	8.0 (4.0–14.0)	12.0 (5.0–17.0)	< 0.001	13.0 (6.75–18.25)	11.0 (5.0–17.0)	7.0 (4.0–14.0)	< 0.001
Patient Health Questionnaire-9 (depression)	9 (4.0–14.0)	8.0 (4.0–13.0)	10.0 (6.0–16.0)	< 0.001	6.0 (3.0–11.25)	8.0 (4.0–12.75)	10.0 (5.0–16.0)	0.006	9.0 (4.0–13.25)	9.0 (5.0–16.0)	0.065	7.0 (3.0–11.0)	10.0 (5.0–15.0)	< 0.001	10.0 (6.0–15.25)	9.0 (4.0–15.0)	6.0 (3.0–10.0)	< 0.001
Kessler (distress)	8 (4.0–12.0)	7.0 (3.0–11.0)	9.0 (5.014.0)	0.002	7.5 (2.0–11.0)	7.0 (3.0–11.0)	8.0 (5.0–13.0)	0.002	7.0 (3.0–11.0)	9.0 (5.0–16.0)	0.002	5.0 (2.0–9.0)	8.5 (4.75–13.0)	< 0.001	8.5 (5.0–12.0)	8.0 (3.0–13.0)	6.0 (3.0–10.0)	0.002

The total median scores (IQR) for each of the depression, anxiety, insomnia, and psychological distress scales were nine (4.0–14.0), 6.0 (3.0–10.0), 11.0 (5.0–7.0), and eight (4.0–12.0), respectively. Female students, those living in locations with a high prevalence of COVID-19 cases, confined for more than 25 days, with a psychiatric consult history, and a preclinical level of studies had higher scores in all four scales. Accordingly, median anxiety scores for those confined in areas with low versus high prevalence of COVID-19 cases were six (3.0–9.0) versus seven (4.0–12.0), *P* < 0.001; median insomnia scores according to preclinical, early, and late clinical level of studies were 13.0 (6.75–18.25), 11.0 (5.0–17.0), and 7.0 (4.0–14.0), *P* < 0.001, respectively; median depression scores for male versus female respondents were 7.0 (3.0–11.0) and 10.0 (5.0–15.0), *P* < 0.001, respectively; and median psychological distress scores for those with or without a history of psychiatric consult were 9.0 (5.0–16.0) and 7.0 (3.0–11.0), *P* = 0.002, respectively (Table 3).

### Risk factors of mental health outcomes.

The results of the ordinal multivariate analysis of factors associated with anxiety during the COVID-19 crisis are presented in Table 4. Significant factors from the univariate analysis were included in the ordered logistic regression analysis. In the model test, *P* < 0.05 indicated that the OR value of at least one variable is statistically significant.

**Table 4:** Risk factors for mental health outcomes by multivariable logistic analysis

Variable	No of severe cases/No of total cases (%)	Odds ratio (95% CI)	*P*-value
GAD-7, anxiety			
Region			
High prevalence of COVID cases (Casa-Marrakech)	29/180 (16.1)	1.628 (1.090–2.432)	0.017
Low prevalence of COVID cases	25/365 (6.8)	1 (Reference)	–
Confinement (days)			
≤ 25	23/286 (8.04)	1 (Reference)	–
> 25	31/263 (11.7)	1.138 (0.851–1.522)	0.384
Gender			
Male	9/143 (6.2)	1 (Reference)	–
Female	45/406 (11.08)	1.653 (1.020–2.679)	0.042
History of psychiatric consult			
No	31/442 (7.01)	1 (Reference)	–
Yes	23/107 (21.4)	1.684 (1.062–2.669)	0.027
Insomnia Severity Index, insomnia			
Region			
High prevalence of COVID cases (Casa-Marrakech)	18/180 (10)	1.370 (0.931–2.018)	0.111
Low prevalence of COVID cases	21/365 (5.7)	1 (Reference)	
Confinement (days)			
≤ 25	15/286 (5.2)	–	–
> 25	24/263 (9.1)	1.278 (0.955–1.709)	0.099
Gender			
Male	4/143 (2.7)		
Female	35/406 (8.6)	1.830 (1.176–2.847)	0.007
Study level			
Preclinical	16/170 (9.4)	0.720 (0.545–0.949)	0.020
Early clinical	20/261(7.6)	–	–
Late clinical	3/118 (2.5)	–	–
Patient Health Questionnaire-9, depression			
Region			
High prevalence of COVID cases (Casa-Marrakech)	58/180 (32.2)	1.438 (1.002–2.097)	0.05
Low prevalence of COVID cases	75/365 (20.5)	–	–
Confinement (days)			
≤ 25	53/286 (18.5)	–	–
> 25	80/263 (30.4)	1.186 (0.903–1.558)	0.220
Gender			
Male	20/143 (13.9)	–	–
Female	113/406 (27.8)	2.167 (1.435–3.271)	< 0.001
Study level			
Preclinical	50/170 (29.4)	0.679 (0.521–0.885)	0.004
Early clinical	66/261 (25.2)	–	–
Late clinical	17/118 (14.4)	–	–
Kessler, distress			
Region			
High prevalence of COVID cases (Casa-Marrakech)	54/180 (30)	1.211(0.829–1.768)	0.0322
Low prevalence of COVID cases	71/365 (19.4)	–	–
Confinement (days)			
≤ 25	51/286 (17.8)	–	–
> 25	74/263 (28.1)	1.107 (0.847–1.448)	0.455
Gender			
Male	16/143 (11.1)	–	–
Female	109/406 (26.8)	1.994 (1.338–2.972)	0.001
Study level			
Preclinical	39/170 (22.9)	0.650 (0.499–0.847)	0.001
Early clinical	67/261 (25.6)	–	–
Late clinical	19/118 (16.1)	–	–
History of psychiatric consult			
No	90/442 (20.36)	–	–
Yes	35/107 (32.7)	1.440 (0.927–2.236)	0.105

The results demonstrated that female gender was a risk factor for severe symptoms of anxiety (OR: 1.653; 95% CI: 1.020–2.679; *P* = 0.042), depression (OR: 2.167; 95% CI: 1.435–3.271; *P* < 0.001), insomnia (OR: 1.830; 95% CI, 1.176–2.847; *P* = 0.007), and psychological distress (OR: 1.994; 95% CI: 1.338–2.972; *P* = 0.001). Being enrolled at a preclinical level of medical studies is a risk factor for depression (OR: 0.679; 95% CI: 0.521–0.885; *P* = 0.004), insomnia (OR: 0.720; 95% CI: 0.545–0.949; *P* = 0.02), and psychological distress (OR: 0.650; 95% CI: 0.499–0.847; *P* = 0.001). Living in a region of high prevalence of COVID-19 cases on the other hand is a risk factor for severe anxiety (OR: 1.628; 95% CI: 1.090–2.432; *P* = 0.017) and depression (OR: 1.438; 95% CI: 1.002–2.097; *P* = 0.05) (Table 4).

## DISCUSSION

The main goal of this study was to evaluate the psychological condition of medical students during the COVID-19 pandemic and explore factors influencing their mental health. This survey indicates that medical students present a high prevalence of mental health disorders, with 62.3%, 74.6%, 62.6%, and 69% having symptoms of anxiety, depression, insomnia, and psychological distress, respectively. The mental health symptoms were associated with gender (female students), place of residence (living in locations with a high prevalence of COVID-19), confinement for more than 25 days, history of psychiatric consult, and a preclinical level of studies. Furthermore, being a female student, having a preclinical level of studies, and living in a location with a high prevalence of COVID-19 cases were independent risk factors for worse mental health during this pandemic. However, no significant differences according to relationship status, having children, or smoking habits were indicated.

Facing the COVID-19 pandemic, countries implemented containment measures such as quarantine. Despite its scientific basis, quarantine is a stressful situation^28^ which increases psychiatric morbidity, as well as the risk of preexisting mental health problems relapsing.^29^ The negative psychological impact of quarantine is also directly aligned with the confinement period which should be as short as possible.^30^ Nonetheless, the world today faces the threat of a second wave which will certainly reflect on the mental well-being as shown by our results. In Morocco, communication units were implemented by the government^31,32^; however, they have no impact on mental distress detection and/or treatment, and more adaptable politics should be adopted.

On the other hand, the geographic variability of mental health outcomes has previously been discussed following both natural and man-made disasters.^33^ During the COVID-19 pandemic, health workers working in areas with a high prevalence of COVID-19 cases also have a high risk of unfavorable mental health.^13^ In our context, Casablanca and Marrakech were the first two cities with positive cases, accounting for more than 45% of the cases, which may explain the high prevalence of mental disorders in our study.

Previous mental health research outlined female gender as a vulnerability factor for worse mental health and lower psychological well-being.^34^ Similarly, female medical students display significantly higher values for depressiveness, and emotional and cognitive burnout,^35^ which exacerbates in disaster situations.

Regarding the COVID-19 mental health burden on college students in general, a Chinese study using the same GAD-7 assessment scale demonstrated severe levels of anxiety in 0.9% of participants.^36^ By contrast, medical students in our study displayed 10 times more severe anxiety cases. In fact, as students enroll in medical studies, their emotional health significantly worsens during the first year.^37^ Embarking on medical studies implies dealing with academic stress and competition, especially as programs include top performing students, and the failure to achieve previous school performance levels can be a source of distress, self-doubt, and anxiety.^38^ Furthermore, the transition from preclinical to clinical training may be complex, and students may face challenges such as professional socialization difficulties, increased workload, and perceived knowledge deficiencies, also known as the “shock of practice.”^39^ In fact, the transition from preclinical to clinical enrollment in some countries plays an important and even selective role in students’ academic performance, which represents an additional source of stress. In our study, an early level of enrollment was an independent risk factor for mental health symptoms.

Amid the COVID-19 crisis, medical students hold a paradoxical position. As they are “not yet MDs,” medical students are considered as vectors for transmission,^40^ hence the suspension of clerkships and clinical activities by some schools. Opposingly, medical students were urged to participate in the fight and even offered early graduation in other circumstances.^14,41^ This uncertainty may add up to the known mental health distress medical students suffer from, which will not only reflect on their current mental health status but also the incidence of mental health issues such as post traumatic stress disorder (PTSD), anxiety, burnout, and substance use in their future as healthcare professionals.

At present, it is important that medical schools not only care about students’ mental health but also implement strategies to support their understanding.^42^ The poor mental health of medical students has always been universally acknowledged, yet no specific actions were taken. However, the COVID-19 pandemic could give rise to proactive measures supporting the well-being of medical students.

Mental health interventions should be included in the crisis response by destigmatizing psychological problems, encouraging communication, and providing psychological support. Quarantined medical students should be initiated on coping skills and emotional exhaustion management techniques, while opportunities for personal and curricular development should be provided. This is particularly important because the current crisis could represent a great opportunity to nurture students’ leadership in the form of peer mentoring, teaching, and self-directed learning. Furthermore, manageable sources of distress such as the worry about examination modalities and academic difficulties should be dealt with by reassuring students and answering their concerns. On the other hand, the discussion about mental disorders in medical students during this pandemic should not be limited to academic research. Longitudinal follow-up studies are required to track the evolution of these symptoms and measure the long-term impact of the pandemic. Henceforth, medical students and healthcare professionals’ predisposition to mental health disorders should no longer be overlooked. Medical faculties should mandatorily contain a specific unit for student counseling and psychological support, aside from natural or health disasters, and a mental disorder detection system should be implemented, especially for anxiety, depression, insomnia, and distress.

This study has several limitations. First, the data collection relied on volunteer sampling through institutional emails and social media platforms only, which could be at the origin of a self-selection bias. Second, female participants represented around two-thirds of the respondents as it reflects the high presence of female gender in medical education and health care. Third, the study was carried on a 10-day period, and a longitudinal follow-up could show an increase in mental health symptoms. Fourth, the study was conducted in Morocco with a limited number of participants. However, as a high incidence of mental symptoms is described with a relatively low number of cases, projecting the results of our study to countries with a higher incidence of COVID-19 cases will certainly show more alarming results.

Notwithstanding these limitations, our study is the first to address medical students’ situation during the pandemic. It concludes on the profile of students with the highest risk for mental health problems that may need mental health interventions during de-confinement and throughout the resumption of hospital activities and lectures. Moreover, large-scale studies involving medical schools could determine the impact locally, whereas early and long-term follow-up will enable adapted and reactive measures. As the number of infected patients continues to increase, so will the psychological burden and the need to assess the progression of the COVID-19 pandemic’ mental health impact on the general population as well.

## CONCLUSION

More than 65% of medical students have experienced psychological distress because of the COVID-19 outbreak, with some students having higher risks than others as shown by our results. Being a female student, living in locations with a high prevalence of COVID-19, being confined for more than 25 days, having a psychiatric consult history, and a preclinical level of studies were risk factors for medical students experiencing mental health symptoms during the COVID-19 outbreak. These alarming results suggest that medical students require attention, help, and support from society, families, and universities. It is suggested that the government and universities should collaborate to resolve this problem and provide high-quality and timely crisis-oriented psychological services to medical students.

## Supplemental Appendix

Supplemental materials
