# Supplementary material for: Predictive Factors for Impaired Mental Health among Medical Students during the Early Stage of the COVID-19 Pandemic in Morocco
Source: Am J Trop Med Hyg. 2020 Nov 17;104(1):95–102. doi: 10.4269/ajtmh.20-1302 (PMC7790070; doi:10.4269/ajtmh.20-1302)
Supplement: Supplementary file 1 [file tpmd201302.SD1.pdf]

Supplemental Appendix 1: Milestone events during the COVID-19 pandemic in Morocco

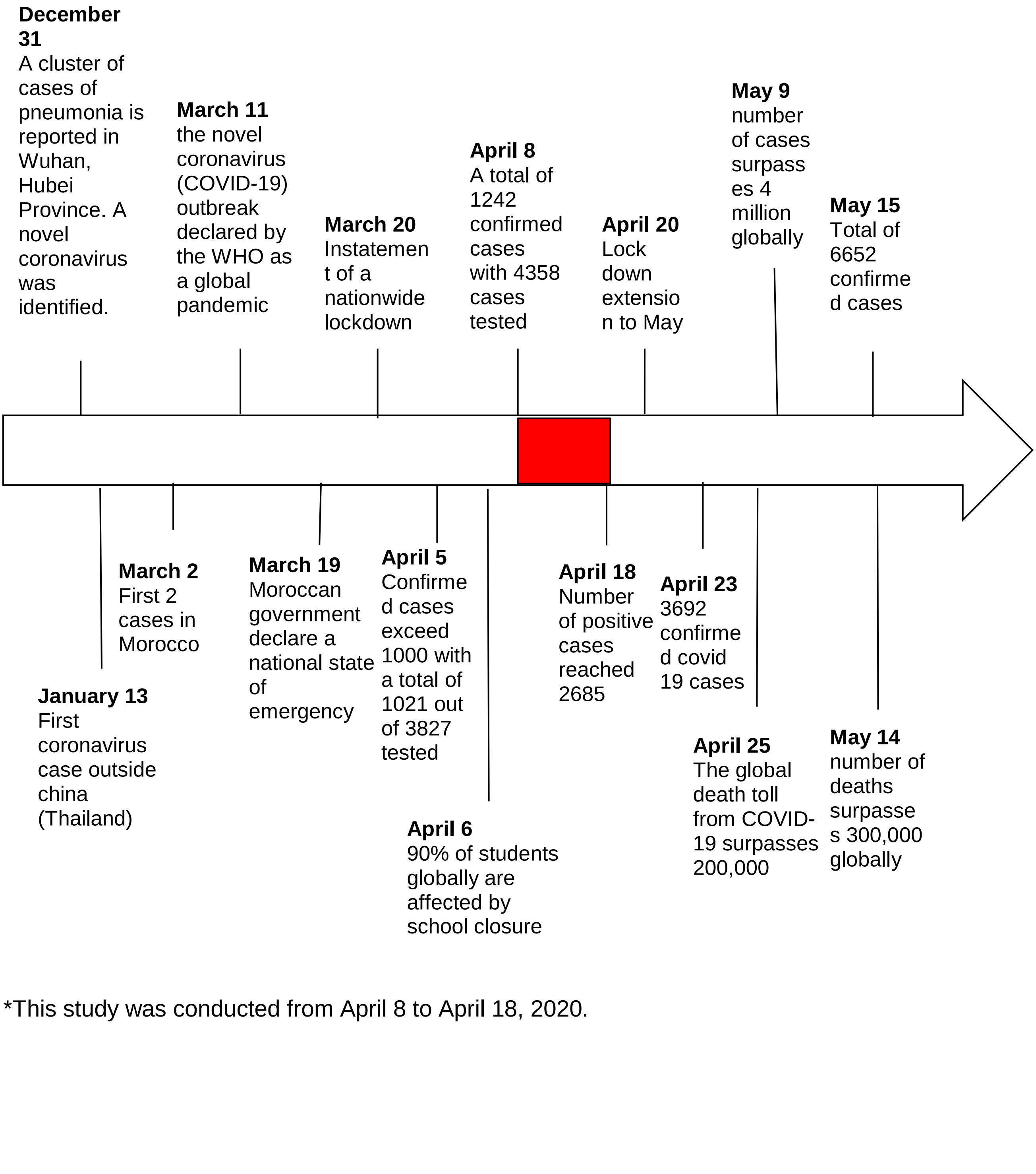

*This study was conducted from April 8 to April 18, 2020.
